# Ultrasonic-Assisted Method for the Preparation of Carbon Nanotube-Graphene/Polydimethylsiloxane Composites with Integrated Thermal Conductivity, Electromagnetic Interference Shielding, and Mechanical Performances

**DOI:** 10.3390/ijms232315007

**Published:** 2022-11-30

**Authors:** Chenglin Li, Zhenzhou Yang, Xiaowen Zhang, Yue Ru, Dali Gao, Daming Wu, Jingyao Sun

**Affiliations:** 1 College of Mechanical and Electrical Engineering, Beijing University of Chemical Technology, Beijing 100029, China; 2 SINOPEC Beijing Research Institute of Chemical Industry, Beijing 100013, China; 3 State Key Laboratory of Organic-Inorganic Composites, Beijing University of Chemical Technology, Beijing 100029, China

**Keywords:** polymer composites, ultrasonic-assisted forced infiltration, thermal conductivity, mechanical properties

## Abstract

Due to the rapid development of the miniaturization and portability of electronic devices, the demand for polymer composites with high thermal conductivity and mechanical flexibility has significantly increased. A carbon nanotube (CNT)-graphene (Gr)/polydimethylsiloxane (PDMS) composite with excellent thermal conductivity and mechanical flexibility is prepared by ultrasonic-assisted forced infiltration (UAFI). When the mass ratio of CNT and Gr reaches 3:1, the thermal conductivity of the CNT-Gr(3:1)/PDMS composite is 4.641 W/(m·K), which is 1619% higher than that of a pure PDMS matrix. In addition, the CNT-Gr(3:1)/PDMS composite also has excellent mechanical properties. The tensile strength and elongation at break of CNT-Gr(3:1)/PDMS composites are 3.29 MPa and 29.40%, respectively. The CNT-Gr/PDMS composite also shows good performance in terms of electromagnetic shielding and thermal stability. The PDMS composites have great potential in the thermal management of electronic devices.

## 1. Introduction

Electronic devices are now rapidly evolving toward high-power and high-energy consumption [1,2]. The miniaturization of device sizes poses a great challenge in terms of heat dissipation. Inadequate internal heat dissipation of electronic components can lead to devices not achieving the expected lifetime and stability. Thermal interface materials (TIM) with good thermal conductivity are therefore urgently needed to improve the heat dissipation capability of devices [3,4,5]. Thermal conductive polymer composites are gaining interest and application as thermal management materials in the microelectronics and energy industries due to their low cost, good chemical resistance, light weight, and excellent mechanical properties [6,7,8,9,10,11]. However, the inherent low thermal conductivity of polymeric materials (0.1 to 0.5 W/(m·K)) limits their engineering applications as thermal management materials [12,13,14,15,16,17]. In order to improve the thermal conductivity of polymeric materials, the addition of thermally conductive fillers to improve the thermal conductivity of polymer composites is the easiest [18,19,20]. High thermal conductive fillers, such as carbon-based fillers (carbon nanotubes (CNTs) [21,22,23], graphene (Gr) [24,25,26,27], carbon fibers [28,29], ceramic particles (boron nitride [30,31,32] and alumina [33,34]), and metal fillers (silver nanowires [35], copper nanoparticles [36], and iron nanoparticles [37])), are introduced into the polymer matrix by the methods described above [38,39,40]. The thermal conductivity of a composite is enhanced by the construction of a continuous thermal conductive network in a polymer matrix through these high-thermal-conductive fillers [41,42].

According to the thermal conductivity path theory, when the volume fraction of a thermal conductivity filler increases to a certain critical value, its thermal conductivity suddenly sharply increases; from an insulator to a conductor, the change range is up to 10 orders of magnitude. This phenomenon is called thermal conductivity seepage, and the corresponding critical value of the thermal conductivity filler volume fraction is called the seepage threshold [43,44]. The mathematical relationship between the percolation threshold and thermal conductivity is as follows [45,46,47]:

$$\lambda_{c}=\lambda_{1}\left[\frac{\lambda_{2}-\left(n-1\right)\lambda_{1}+\left(n-1\right)V_{2}\left(\lambda_{2}-\lambda_{1}\right)}{\lambda_{2}+\left(n-1\right)\lambda_{1}-V_{2}\left(\lambda_{2}-\lambda_{1}\right)}\right]$$

where *λ_c_* is the thermal conductivity of the composite; *λ*_1_ and *λ*_2_ are the thermal conductivities of the continuous phase matrix and dispersed phase particles, respectively; *V*_2_ is the volume percentage of the dispersed phase particles; *n* = *3/ψ*, *ψ* is the sphericity of the particle. Although some researchers tend to believe that the percolation behavior of thermal conductive polymer composites should be similar to that of electrically conductive composites, however, compared with the electrical conductivity, the thermal conductivity of composites increases at a relatively low rate with the increase in thermal conductivity filler content. The most common way to solve this problem is to increase the content of highly thermally conductive fillers in the composite system [48,49]. While higher filler content can improve the thermal conductivity of polymer composites, it can also reduce the processing and mechanical properties of polymer composites.

Many experts have proposed new synthetic and processing methods to prepare polymer composites to solve these problems. Pan et al. [50] developed a composite film based on reduced graphene oxide (rGO) and significantly increased the in-plane thermal conductivity of the composite film from 0.055 W/(m·K) to 0.091 W/(m·K) by introducing CNTs. However, the mutual van der Waals forces of the CNTs cause them to aggregate between the Gr layers, limiting the in-plane thermal conductivity of the Gr-based films. Yu et al. [51] prepared Gr/multiwalled carbon nanotubes (MWCNTs)/polycarbonate composites by using a solution-blending method with a thermal conductivity of 1.39 W/(m·K) at 20% loading (mass fraction) of Gr and MWCNTs. Shao et al. [52] improved the thermal conductivity of polyamide composites to 0.891 W/(m·K) through the synergistic effect of boron nitride nanosheets (1.6 wt%) and Gr (6.8 wt%) in a three-dimensional (3D) thermal conductivity network. Sun et al. [22] prepared natural rubber (NR) as a model matrix and introduced one-dimensional (1D) CNT into a 3D wood-based carbon scaffold (CS) to prepare a 3D interconnected filler network with continuous interconnections. When the content of CNT was 1.67 vol%, the thermal conductivity of the CS/CNT/NR composite was 1.1 W/(m·K), and thermal conductivity enhancement was 444%. Wu et al. [53] prepared an ideal filler structure for a thermal conductive composite by filling aligned graphene nanosheets (GNs) in a continuous network of graphene foam (GF). The GNs/GF/natural rubber composite showed a thermal conductivity enhancement of 8100%, and the thermal conductivity of the composite was enhanced by 1300% for every 1 vol% increase in Gr. Researchers have used the continuous spatial confining method, sol-gel, solution blending, and phase separation to form better thermally conductive networks within the polymer matrix [7,41]. However, these methods involve first blending and then building the thermal conductivity network, whereas UAFI first builds the thermal conductivity network and then introduces it into the matrix. The advantage of this method is that good flexibility can be achieved at high filling levels. Vacuum-assisted infiltration (VAF) is a common method for polymer matrix infiltration using negative vacuum pressure. However, the method has some limitations and is applicable only to the infiltration of low-viscosity polymer matrix and thermal conductive network systems with large gap distances. To solve this problem, we propose an efficient ultrasonic-assisted forced infiltration (UAFI) method.

**Figure 1:**
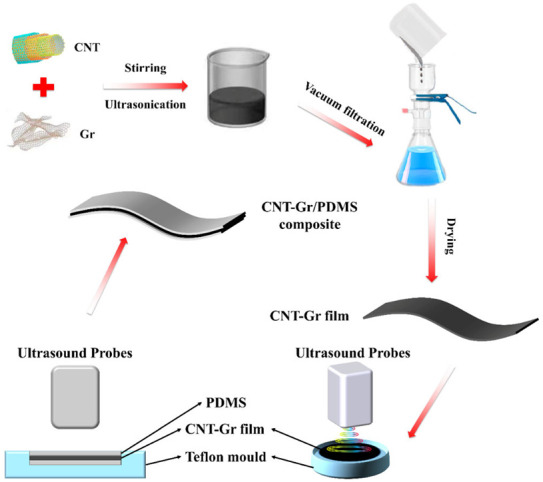
Schematic diagram of the CNT-Gr film and CNT-Gr/PDMS composite preparation process.

In this work, thermosetting polydimethylsiloxane (PDMS) was chosen as the polymer matrix because of its excellent wear resistance, aging resistance, flexibility, and elasticity. One-dimensional CNT and two-dimensional (2D) Gr with high thermal conductivity were chosen as thermal conductive fillers. In addition, PDMS-based composites have good adhesion to different materials, which facilitates their application as a thermal management material. However, the general VAF method cannot meet the requirement of infiltrating the highly viscous PDMS matrix into the dense CNT-Gr films prepared by VAF. Therefore, we designed an UAFI method to prepare CNT-Gr/PDMS composites (as shown in Figure 1). Ultrasonic welding machines with high power and high frequency (2 kW and 20 kHz) were used as ultrasonic sources for forced infiltration. The effects of different mass ratios of CNT to Gr (3:1, 1:1, and 1:3, respectively) on the morphology, thermal conductivity, and mechanical properties of CNT-Gr/PDMS composites were investigated in detail. In addition, a comprehensive evaluation of the thermal management properties of CNT-Gr/PDMS composites was carried out. The results show that the CNT-Gr/PDMS composite prepared by UAFI is a promising, excellent thermal management material.

## 2. Results and Discussion

**Figure 2:**
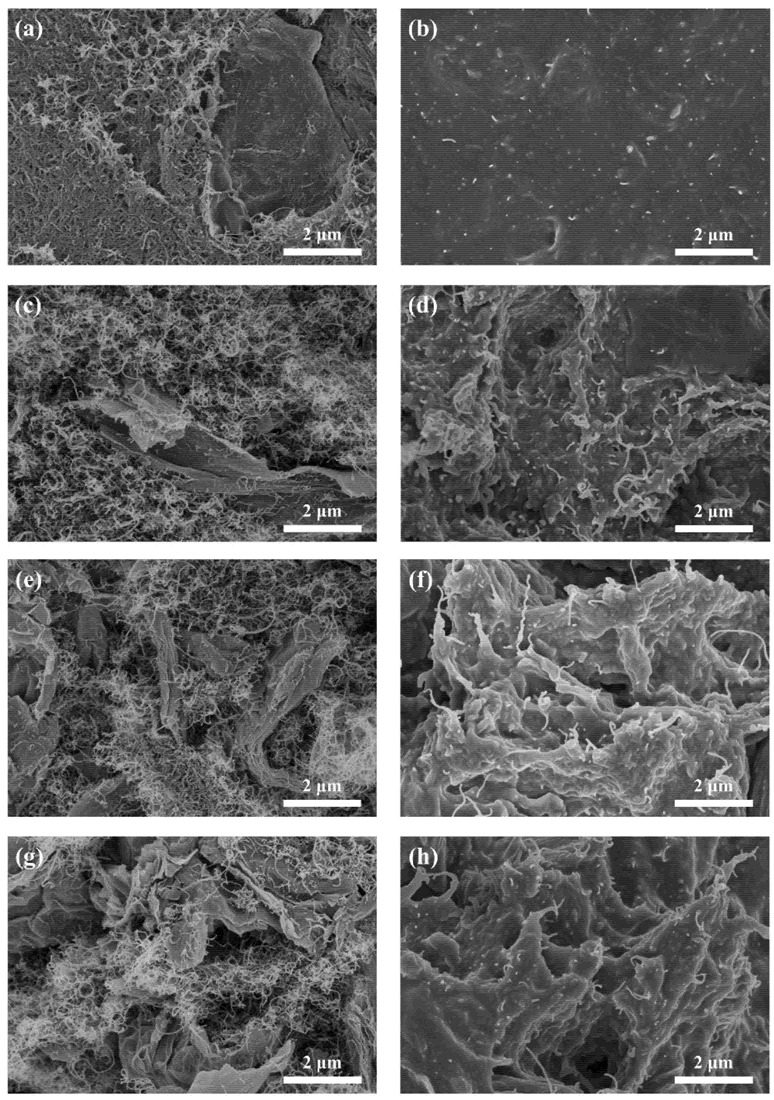
In-plane morphologies of (**a**) the CNT-Gr(3:1) film and (**b**) the CNT-Gr(3:1)/PDMS composites. The through-plane morphologies of (**c**) the CNT-Gr(3:1) film, (**d**) the CNT-Gr(3:1)/PDMS composites, (**e**) the CNT-Gr(1:1) film, (**f**) the CNT-Gr(1:1)/PDMS composites, (**g**) the CNT-Gr(1:3) film, and (**h**) the CNT-Gr(1:3)/PDMS composites.

Figure 2 shows SEM images of the composites with different CNT:Gr mass ratios. As shown in Figure 2a, the CNT-Gr(3:1) films were prepared by VAF in in-plane morphologies. From the diagram, we can observe that there is a large amount of CNT and a small amount of 2D structure in Gr. CNT and Gr are tightly bonded to form an excellent electrically conductive and thermal conductive network. After the CNT-Gr film was infiltrated with PDMS by using a UAFI method, the surface of the CNT-Gr(3:1)/PDMS composite was covered with a flat PDMS layer (as shown in Figure 2b). In addition, a small amount of CNT can be observed on the surface of the CNT-Gr(3:1)/PDMS composite sample. However, the 2D structure Gr is wrapped in PDMS, so that Gr is no longer observed on the surface. Figure 2c illustrates the through-plane morphology of the CNT-Gr(3:1) film, where it can be observed that a laminar structure of Gr is formed. The thermal conductive network of the film is formed by the combination of CNT and Gr filler. The through-plane morphology of the CNT-Gr(3:1)/PDMS composite after infiltration with PDMS is shown in Figure 2d. It can be seen that the small gaps inside the network are filled with PDMS, allowing the thermal conductive network to be further compacted. As the CNT:Gr mass ratio increases, it is evident that the Gr content inside the network increases (shown in Figure 2e,g). However, the internal network defects increase, leading to an increase in the interfacial thermal resistance of the composite. Therefore, in order to build a good thermal conductivity network and reduce internal thermal resistance, the synergistic effect of CNT and Gr is needed. CNT is used to fill the gaps between Gr to reduce internal thermal resistance. As shown in Figure 2f,h, after the CNT-Gr films are infiltrated with PDMS, some of the gaps are filled by PDMS. Compared with the in-plane morphology of the CNT-Gr(3:1)/PDMS composites, the CNT-Gr(1:1)/PDMS and CNT-Gr(1:3)/PDMS composites formed more defects in the thermal conductive network and so resulted in lower thermal conductivity in the samples.

**Figure 3:**
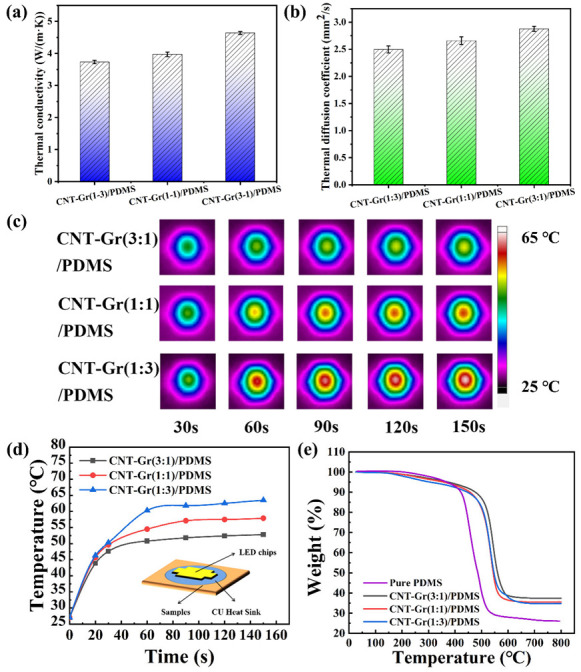
(**a**) The thermal conductivities and (**b**) the thermal diffusion coefficients of the CNT-Gr/PDMS composites as a function of the CNT:Gr mass ratio. (**c**) The thermal infrared images of LED chips using different CNT-Gr/PDMS composites as thermal management materials. (**d**) The plot of surface temperature versus time for LED chips with different CNT-Gr/PDMS composites as TIM. The inset in Figure 3d shows a schematic diagram of the thermal properties of the CNT-Gr/PDMS composite tested with LED chips. (**e**) TGA curves of the CNT-GR /PDMS composites with CNT and Gr fillers mixed at various ratios: 3:1, 1:1, and 1:3.

The thermal diffusion coefficient and thermal conductivity of CNT-Gr/PDMS composites as a function of the CNT:Gr mass ratio are shown in Figure 3 (specific data are shown in Table 1). The thermal diffusion coefficient of CNT-Gr/PDMS composites increased with increasing CNT to Gr mass ratio from 1:3 to 3:1. When the mass ratio of CNT to Gr reached 3:1, the thermal diffusion coefficient of CNT-Gr(3:1)/PDMS composites reached 2.877 mm^2^/s, 8.2% and 15.2% higher than that of CNT-Gr(1:1)/PDMS composites and CNT-Gr(1:3)/PDMS composites, respectively. The thermal conductivity of CNT-Gr/PDMS composites also increased with increasing CNT:Gr mass ratio. When the mass ratio of CNT to Gr reached 3:1, the thermal conductivity of the CNT-Gr(3:1)/PDMS composite reached 4.641 W/(m·K): 16.8% and 24.3% higher than that of the CNT-Gr(1:1)/PDMS composite and CNT-Gr(1:3)/PDMS composite, respectively. This is because CNT-Gr(3:1)/PDMS composites had a better internal thermal conductivity network than the other CNT-Gr/PDMS composites. When the CNT to Gr mass ratio was 3:1, the CNT-Gr(3:1) films had fewer internal defects and formed a denser thermal conductive network. After infiltration of PDMS, the denseness of the thermal conductive network was further improved.

The effect of the filler mass ratio CNT:Gr on the thermal conductivity of the PDMS composite was further analyzed by recording the temperature change on the surface of the LED chip with an infrared thermal imaging camera. Different CNT-Gr/PDMS composites were used as TIM connected between the LED chip (10 W) and the Cu heat sink (as shown in Figure 3d). An infrared image of the surface temperature change of the chip from 0 to 150 s is shown in Figure 3c. It can be clearly seen that the surface heating rate of the chip was slower than that of other CNT-Gr/PDMS composites when using the CNT-Gr(3:1)/PDMS composite as the TIM. Figure 3d shows the temperature variation curve of different CNT-Gr/PDMS composites after heating the LED chip for 150 s. After 150 s of heating to stabilize, the surface temperature of the chip using the CNT-Gr(3:1)/PDMS composite as the TIM reached ~52.8 °C, lower than the surface stabilization temperature of the CNT-Gr(1:1)/PDMS and CNT-Gr(1:3)/PDMS composites. At the same filler content (12 wt%), the thermal conductivity of our CNT-Gr(3:1)/PDMS composites prepared by the UAFI method is higher than that of hexagonal boron nitride/multiwall carbon nanotubes/PDMS prepared by the continuous space-confining method [7]. The above results show that the CNT-Gr(3:1)/PDMS composites prepared by the UAFI method have the best thermal conductivity and thermal management properties.

The thermal stability of the CNT-Gr/PDMS composites was analyzed by thermal gravimetric analysis (TGA) (as shown in Figure 3e). In order to better evaluate the thermal stability of the different PDMS composites, the heat-resistance index (*T_HRI_*) of the PDMS composites was calculated according to the following equation:

$$T_{HRI}=\left[T_{5}+\left(T_{30}-T_{5}\right)\times 0.6\right]\times 0.49$$

where *T_30_* and *T_5_* are the decomposition temperatures at 30% and 5% weight loss, respectively. As can be observed in Table 2, the *T_HRI_* of the CNT-Gr(3:1)/PDMS composite was 231.05 °C, higher than that of the CNT-Gr(1:1)/PDMS and CNT-Gr(3:1)/PDMS composites (223.55 °C and 213.82 °C). This result indicates that the CNT-Gr(3:1)/PDMS composite had significantly improved thermal stability compared with other composites. This is because as the CNT to Gr mass ratio increases, the thermal conductive network within the film becomes denser and has fewer internal defects, enhancing the thermal stability of the CNT-Gr(3:1)/PDMS composite.

Assuming that the mass fraction of CNT and Gr in the CNT-Gr/PDMS composite is *X* and that the mass fraction of PDMS is *Y*, the residuals of each sample at 800 °C are given in Figure 3e. A binary equation based on the quantitative relationship is presented

$$\left\{\begin{array}{c} X+Y=1\\ X+bY=c \end{array}\right.$$

where *b* is the residual amount of pure PDMS at 800 °C, 28.80 wt%; *c* is the residual amount at 800 °C in the different CNT-Gr/PDMS composites (as shown in Table 3).

Using TGA, it is possible to calculate the detailed mass fractions of filler and PDMS matrix for CNT-Gr/PDMS composites. The TGA curve of pure PDMS (as shown in Figure 3e) is used as a reference line to calculate the mass fraction of the different composites.

The mass fractions of CNT, Gr filler, and the PDMS matrix in the different CNT-Gr/PDMS composites are given in Table 4. The CNT and Gr fillers in the CNT-Gr(3:1)/PDMS composites totaled 12.20 wt%, higher than in the CNT-Gr(1:1)/PDMS (10.00 wt%) and CNT-Gr(1:3)/PDMS (8.44 wt%) composites. This result further explains the higher λ of the CNT-Gr(3:1)/PDMS composite compared with that of the other composites. In addition, the mass ratios of CNT and Gr were 3:1, 1:1, and 1:3. The content of CNT and Gr fillers in each PDMS composite was determined.

The electromagnetic interference (EMI) shielding effect is divided into three components, namely absorption (*SE_A_*), reflection (*SE_R_*), and multiple reflection (*SE_M_*). The formula for calculating EMI SE is as follows [54,55,56]:


$$SE_{T}=SE_{A}+SE_{R}$$

The *SE_T_*, *SE_A_*, and *SE_R_* values are determined by the parameters of S (measured by the vector network analyzer) and are calculated as follows:

$$R=10^{\left(S_{11}/10\right)},\;T=10^{\left(S_{21}/10\right)},\;A=1-R-T$$

$$SE_{R}=-10log\left(1-R\right),\;SE_{A}=-10log\left(T/\left(1-R\right)\right)$$

$$SE_{T}=SE_{R}+SE_{A}=-10log\;T$$

where *R* is the reflection coefficient*, A* is the absorption coefficient, and *T* is the transmission coefficient.

**Figure 4:**
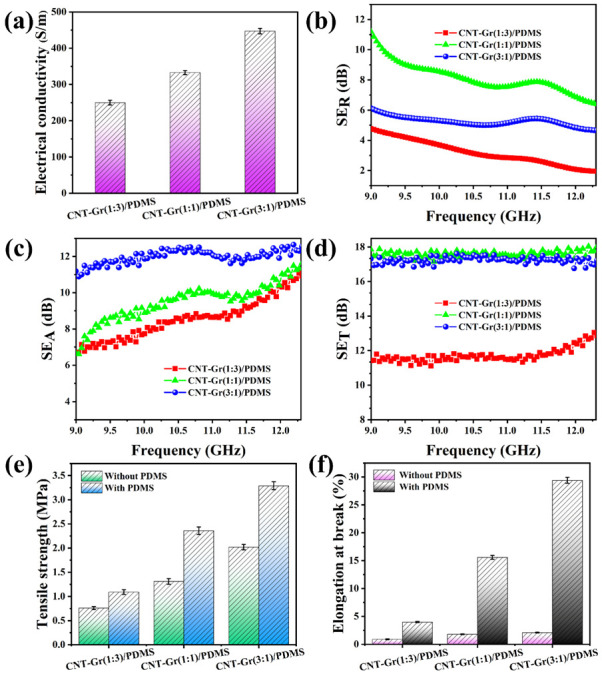
Plots of (**a**) electric conductivity, (**b**) EMI SE_R_, (**c**) SE_A_, and (**d**) SE_T_ of PDMS composites with different CNT:Gr mass ratios. (**e**) The tensile strength and (**f**) the elongation at the break of different CNT-Gr/PDMS composite samples.

We tested the EMI shielding performance *SE_T_*, *SE_R_*, and *SE_A_* of PDMS composites in the frequency range of 8.2–12.4 GHz (X-band). The ratio of CNT to Gr in CNT-Gr/PDMS composites is one of the most important factors affecting the EMI shielding performance of their composites. This factor is demonstrated in this study. As the CNT:Gr mass ratio increased, the EMI *SE_T_* of the PDMS composite first increased and then decreased (as shown in Figure 4d). At a frequency of 9 GHz, the CNT:Gr(1:1)/PDMS (17.86 dB) composite had the highest EMI *SE_T_* value, higher than those of the CNT:Gr(1:3)/PDMS (11.41 dB) and CNT:Gr(3:1)/PDMS (17.31 dB) composites. CNT:Gr(1:3)/PDMS, CNT:Gr(1:1)/PDMS, and CNT:Gr(3:1)/PDMS composites are available in thicknesses of 0.16 mm, 0.14 mm, and 0.13 mm, respectively. The electric conductivity of the CNT-Gr/PDMS composites is shown in Figure 4a. With the increase in the CNT:Gr mass ratio, the conductivity of CNT-Gr/PDMS composite material also increases.

Figure 4 illustrates the mechanical properties of CNT-Gr/PDMS composites as a function of CNT to Gr mass ratio. The tensile strength and elongation at break of the CNT-Gr/PDMS composites show an upward trend with the increase in the mass ratio of CNT to Gr from 1:3 to 3:1. When the mass ratio of CNT to Gr reaches 3:1, the tensile strength and elongation at break of the CNT-Gr(3:1)/PDMS composite reach a maximum of 3.29 MPa and 29.40%, respectively. As the Gr content increases, the tensile strength and elongation at the break of the CNT-Gr/PDMS composites decrease. The reasons can be explained by the increased Gr content, which caused a large number of defects in the internal structure of the CNT-Gr/PDMS composite (as shown in Figure 2e,g). The tensile strength of the CNT-Gr(1:3)/PDMS composite remained at a high level of 1.09 MPa, reflecting the excellent mechanical properties of the CNT-Gr/PDMS composite. In this study, the tensile strength and elongation at break of the CNT-Gr/PDMS composites showed highly consistent trends and the excellent microstructure obtained by infiltrating PDMS into the CNT-Gr films through UAFI.

**Figure 5:**
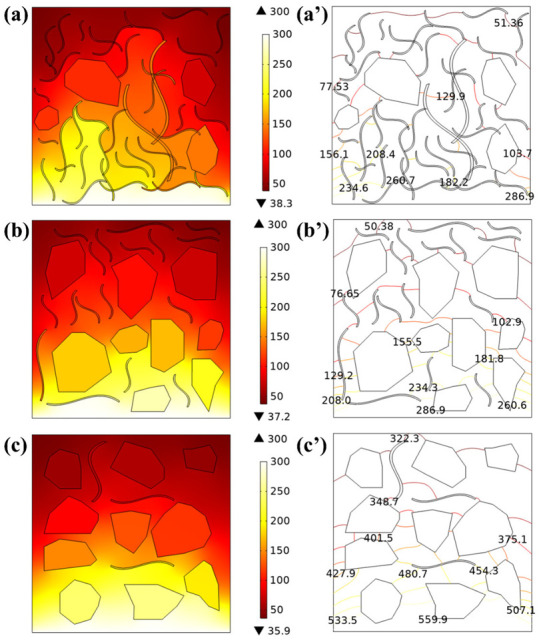
Simulation results of surface temperatures and isotherms of PDMS composites with different CNT:Gr mass ratios. (**a**,**a’**) CNT-Gr(3:1)/PDMS composites, (**b**,**b’**) CNT-Gr(1:1)/PDMS composites, and (**c**,**c’**) CNT-Gr(1:3)/PDMS composites.

The internal thermal conductivity network model of the PDMS composite was constructed by using COMSOL (COMSOL Co., Ltd., Stockholm, Sweden) simulation software on the basis of the composition structure of the CNTs and Gr inside the CNT-Gr/PDMS composite in SEM images, and simulations were carried out using the heat transfer module (as shown in Figure 5). A heat source of constant temperature (300 °C) was applied to the bottom of the CNT-Gr/PDMS composite model. Figure 5a–c show the simulation results for CNT-Gr(3:1)/PDMS, CNT-Gr(1:1)/PDMS, and CNT-Gr(1:3)/PDMS composites, respectively. The simulation results show that the area of thermal diffusion decreases as the CNT content decreases. This is consistent with the results of previous thermal conductivity tests. As shown in Figure 5, CNT-Gr(3:1)/PDMS composites have a tight bond between the CNTs, allowing for better heat transfer and diffusion at the interface. The above results show that the CNT-Gr(3:1)/PDMS composite has both excellent thermal conductivity and good heat dissipation capabilities. The simulation results for the CNT-Gr/PDMS composite isotherms are shown in Figure 5a’–c’.

## 3. Materials and Methods

### 3.1. Materials

The carbon nanotubes (CNTs) with a diameter of 30–70 nm and a length greater than 2 μm in length were supplied by the Russian Nanotechnology Centre Tambov Co., Ltd. (Moscow, Russia). The graphene (Gr) was purchased by Congzhou Sixth Element Materials Technology Co., Ltd. (Changzhou, China). The sodium dodecyl sulfate (SDS) was supplied by Tianjin Beichen Founder Reagent Factory (Tianjin, China). Polydimethylsiloxane (PDMS, SYLGAR 184), with a density of 1.03 g/cm^3^ and a thermal conductivity of 0.27 W/(m⋅K), was purchased from Dow Corning, (Midland, MI, USA). LED chips (10 W) were purchased from Ruijie Co., Ltd. (Fuzhou, Fujian).

### 3.2. Preparation of the CNT-Gr/PDMS Thermally Conductive Composites

The CNT-Gr films were first prepared via VAF (as shown in Figure 1). Firstly, 1 g of filler (CNT and Gr) and 2 g of SDS were added to deionized water to obtain 400 mL of dispersion, which was stirred with a magnetic stirrer (SY-DF3-1A, Shanghai Shangpu Instrument Equipment Co., Ltd., Shanghai, China) for 10 min. The dispersions had different CNT:Gr mass ratios of 3:1, 1:1, and 1:3. Subsequently, the three dispersed solutions were separately sonicated for 10 h using a probe sonicator to obtain uniformly dispersed CNT-Gr solutions. The three CNT-Gr solutions were filtered through polytetrafluoroethylene (PTFE) membranes with a diameter of 47 mm and a pore size of 0.22 μm. CNT-Gr films could be obtained by stripping the PTFE film. The films were named CNT-Gr(3:1), CNT-Gr(1:1), and CNT-Gr(1:3) according to the mass ratio of CNT:Gr. Then, the CNT-Gr/PDMS composite was prepared by UAFI (as shown in Figure 1). Firstly, PDMS was mixed with a curing agent at a mass ratio of 10:1 at room temperature and dried in a vacuum drying chamber for 15 min to remove the air bubbles from the PDMS. A sandwich structure of liquid PDMS, CNT-Gr film, and liquid PDMS was constructed layer-by-layer in a PTFE mold (as shown in Figure 1). The setting of the ultrasonic parameters had a large influence on the thermal conductivity of the PDMS composite. According to the preliminary experiment of high-power and high-frequency ultrasonic welder machines (2 kW, 20 kHz), too large ultrasonic amplitude and too long processing time will damage the CNT-Gr film [42]. Therefore, the parameters of the ultrasonic welding machine were selected as an ultrasonic amplitude of 50%, a frequency of 20 kHz, and a processing time of 2.3 s. The ultrasonic equipment was provided by Shenzhen Jinliwei Technology Co., Ltd., Shenzhen, China. The PDMS matrix was infiltrated into CNT-Gr films by UAFI, which were named CNT-Gr(3:1)/PDMS composites, CNT-Gr(1:1)/PDMS composites, and CNT-Gr(1:3)/PDMS composites according to the mass ratio of CNT:Gr.

### 3.3. Characterization

Scanning electron microscopy (SEM) (S4700, Hitachi, Japan) was used to observe the morphology of the composite samples and to analyze the results of the infiltration of the PDMS matrix into the CNT-Gr films. The thermal conductivity of the composite samples was measured at room temperature (25 °C) and calculated by the equation $K\;=\alpha \cdot C_{p}\cdot \rho$, where α (mm^2^/s) is the planar thermal diffusion coefficient, which we measured by the laser flash technique based on a Netzsch system (LFA 467, NETZSCH, Selb, Germany). Specific heat $C_{p}$ (J/(g⋅K)) was measured using a DSC Pyris 1 (PerkinElmer, Waltham, MA, USA). ρ (g/cm^3^) is the density of the final sample (shown in the detailed data in Table 1). Video of the thermal management analysis was recorded using an infrared thermal imager (TIS60, Fluke, Everett, WA, USA), and the video was inputted into SmartView software(Fluke, Everett, WA, USA) for further analysis. The mechanical properties of the CNT-Gr/PDMS composite samples were tested using a universal testing machine (UTM-1422, Chengde Jinjian Testing Instruments Co., Ltd., Chengde, China).

## 4. Conclusions

The thermal diffusion coefficient and thermal conductivity of the CNT-Gr/PDMS composites increase with increasing the mass ratio of CNT to Gr. The corresponding thermal conductivities are 4.641, 3.974, and 3.733 W/(m·K) for CNT to Gr mass ratios of 1:3, 1:1, and 3:1, respectively. The maximum tensile strength and elongation at break of the CNT-Gr/PDMS composites increase with increasing mass ratio of CNT to Gr. The corresponding maximum tensile strengths are 1.09, 2.36, and 3.29 MPa, and the elongations at break are 4.00%, 15.60%, and 29.40% for CNT to Gr mass ratios of 1:3, 1:1, and 3:1, respectively. The CNT-Gr/PDMS composite also has good electromagnetic shielding properties and thermal stability. CNT-Gr(3:1)/PDMS composites prepared by ultrasonic-assisted forced infiltration have the best thermal conductivity and mechanical properties and have great potential for thermal management applications.

## Figures and Tables

**Table 1:** λ, α, *C_P_*, and *ρ* of PDMS samples with different CNT and Gr filling ratios.

Composites	*λ*	α	*C_P_*	*ρ*
CNT:Gr(3:1)/PDMS	4.641 ± 0.043	2.877 ± 0.063	1.330	1.213
CNT:Gr(1:1)/PDMS	3.974 ± 0.074	2.659 ± 0.071	1.260	1.186
CNT:Gr(1:3)/PDMS	3.733 ± 0.053	2.498 ± 0.046	1.320	1.132

**Table 2:** Heat-resistance index of different CNT-Gr/PDMS composites.

Sample	Weight-Loss Temperature (°C)		
	*T*_5_	*T*_30_	*T_HRI_*
CNT-Gr(3:1)/PDMS	373.33	537.00	231.05
CNT-Gr(1:1)/PDMS	356.33	522.83	223.55
CNT-Gr(1:3)/PDMS	305.17	523.83	213.82

**Table 3:** The *c* values of different CNT-Gr/PDMS composites.

Samples	*c* Value
CNT-Gr(3:1)/PDMS	37.48%
CNT-Gr(1:1)/PDMS	35.60%
CNT-Gr(1:3)/PDMS	34.80%

**Table 4:** The mass fractions of fillers and polymer matrix in each CNT-Gr/PDMS.

	CNT-Gr(3:1)/PDMS	CNT-Gr(1:1)/PDMS	CNT-Gr(1:3)/PDMS
CNT	9.15	5.00	2.11
Gr	3.05	5.00	6.33
PDMS	87.80	90.0	91.56

## Data Availability

The data presented in this study are available on request from the corresponding author.
